# Persistent Low-Grade Squamous Intraepithelial Lesions and the Risk of Overtreatment: Evidence from Long-Term Active Surveillance

**DOI:** 10.3390/cancers18060921

**Published:** 2026-03-12

**Authors:** Maria Teresa Bruno, Alessia Pagana, Ilenia Concetta Palermo, Maria Fiore, Roberta Siena, Carla Lo Giudice, Antonino Giovanni Cavallaro, Liliana Mereu

**Affiliations:** 1 Department of General Surgery and Medical Surgery Specialties, Gynecological Clinic Policlinico Rodolico, University of Catania, 95123 Catania, Italy; 2 Department of Biomedical and Biotechnological Sciences, Clinical Virology, University of Catania, 95123 Catania, Italy; 3 Department of Medical, Surgical Sciences and Advanced Technologies G.F. Ingrassia, University of Catania, 95123 Catania, Italy

**Keywords:** LSIL, active surveillance, overtreatment

## Abstract

Background: Low-grade squamous intraepithelial lesions (LSIL/CIN1) are among the most common abnormalities detected in cervical cancer screening, particularly in the era of primary HPV testing. Despite their low oncogenic potential, persistence of LSIL is still frequently interpreted as a marker of progression risk, often leading to intensified surveillance or excisional treatment and raising concerns about overtreatment. Methods: We conducted a retrospective cohort study including women with persistent LSIL managed through active surveillance in a real-world clinical setting. Time-to-event analyses were performed using Kaplan–Meier estimates to evaluate CIN3-free survival. The association between HPV16 status and progression to CIN3 was assessed using Cox proportional hazards models. The proportional hazards assumption was tested using Schoenfeld residuals, and a prespecified landmark analysis at 24 months was performed to explore potential time-dependent effects. Results: A total of 82 women were included, with a median follow-up of 48 months (range 24–78). Progression to histologically confirmed CIN3 occurred in 7 cases (8.5%). Most CIN3 events (6/7, 85.7%) were diagnosed within the first 24 months of surveillance, followed by a plateau in long-term CIN3-free survival. HPV16-positive women showed a higher incidence of CIN3 compared with non-HPV16 women. In Cox regression analysis, HPV16 positivity showed a higher estimated hazard of CIN3; however, the association did not reach statistical significance and confidence intervals were wide, reflecting the limited number of outcome events. Conclusions: In women with persistent LSIL/managed by active surveillance, progression to CIN3 was uncommon and predominantly occurred early during follow-up. Long-term persistence of LSIL alone did not confer a sustained excess risk of high-grade disease. These findings support a risk-based management approach and suggest that excisional treatment based solely on LSIL persistence may contribute to overtreatment without meaningful oncologic benefit. However, given the limited number of events, the results should be interpreted cautiously and primarily as descriptive evidence.

## Simple Summary

Persistent low-grade squamous intraepithelial lesions (LSIL) are frequently managed aggressively due to concerns about progression, raising the risk of overtreatment. In this retrospective cohort study of women undergoing active surveillance for persistent LSIL/CIN1, progression to CIN3 was uncommon and occurred predominantly within the first 24 months of follow-up. HPV16 positivity was associated with an increased early risk of CIN3, while long-term persistence of LSIL alone did not confer a sustained excess risk. These findings support a risk-based management strategy and suggest that excisional treatment based solely on LSIL persistence may lead to unnecessary interventions without oncologic benefit.

## 1. Introduction

Cervical cancer remains a major global public health concern and represents the fourth most common cancer among women worldwide, with approximately 660,000 new cases and 350,000 deaths reported in 2022 [1]. Persistent infection with oncogenic human papillomavirus (HPV) is the necessary cause of virtually all cervical cancers and underlies the development of both low-grade and high-grade cervical lesions [2]. The primary aim of cervical cancer screening is therefore to prevent invasive disease through the timely detection and treatment of high-grade cervical precancer (CIN2+) before malignant transformation occurs [3].

With the widespread implementation of HPV-based primary screening, detection of transient HPV infections and low-grade abnormalities has increased substantially [4]. As a consequence, distinguishing lesions with low malignant potential from those associated with clinically relevant risk has become increasingly important.

Low-grade squamous intraepithelial lesion (LSIL) represents one of the most frequent abnormal findings in cervical cancer screening programs, particularly in the era of HPV-based primary screening, which has led to an increase in the identification of persistent infections and low-grade cytological abnormalities [4]. HPV infection can involve the entire lower genital tract, including the vagina and vulva due to the “field effect” [5]. LSIL is generally considered to be the expression of a productive HPV infection, characterized by viral replication, a high probability of spontaneous regression and an intrinsically low risk of progression to high-grade lesions [6,7]. Despite this favorable biological profile, the clinical management of persistent LSIL remains a matter of debate. In clinical practice, the persistence of LSIL over time is frequently used as a criterion of increased oncological risk, leading to intensified follow-up or, worse, excisional treatments even in the absence of high-grade histological evidence. This approach reflects a traditional conceptual model that interprets the progression from LSIL to HSIL as a linear continuum [8]; however, increasingly robust biological, virological, and histopathological evidence calls this paradigm into question [9,10,11].

It is now widely recognized that LSIL/CIN1 and CIN3 represent biologically distinct entities, as fully described by Schiffman and Wentzensen [12]. Low-grade lesions are generally associated with productive infection, whereas high-grade lesions reflect a transforming infection with true oncogenic potential. In this context, the persistence of LSIL does not necessarily imply disease progression, and the appearance of high-grade lesions can occur de novo, independently of a pre-existing low-grade lesion [13,14].

At the same time, the clinical consequences of unnecessary excisional treatment are well documented. Numerous studies have demonstrated an increased risk of adverse obstetric outcomes and gynecological morbidity associated with cervical excisional procedures, underscoring the importance of avoiding unnecessary interventions in low-risk women [15,16]. Therefore, balancing oncological safety and iatrogenic harm is a crucial element in the management of persistent LSIL.

The most recent risk-based guidelines, ASCCP, 2019 and ESGO, 2023, have emphasized the need to move beyond a decision-making approach based solely on cytological results or lesion duration, promoting a dynamic and individualized assessment of the risk of clinically relevant progression [17,18]. However, to date, few studies have specifically quantified the true risk of CIN3+ in women with persistent LSIL managed in contemporary clinical practice and the true extent of associated overtreatment. A recent nationwide Norwegian study including over 26,000 women with histologically confirmed CIN1 reported long-term data on progression to CIN2+ and CIN3+, highlighting that although overall progression risk remains limited, treatment rates are substantial in routine practice. These findings further underscore the need to refine risk stratification in low-grade disease [19].

In light of these considerations, the aim of this study is to evaluate the natural history of persistent HPV-positive LSIL in a cohort undergoing active surveillance. This study analyzes the incidence of high-grade cervical lesions in women with persistent LSIL and determines whether persistence, in the absence of additional risk factors, represents an appropriate justification for excisional treatment. By applying a risk-based approach to real-world clinical data, this study aims to contribute to more appropriate and less iatrogenic management of persistent LSIL.

## 2. Materials and Methods

### 2.1. Study Design

A retrospective, single-center, observational study based on data collected in clinical practice was conducted. The aim was to describe the natural history of persistent low-grade squamous intraepithelial lesion (LSIL) in HPV-positive women undergoing active surveillance and to evaluate the incidence and temporal distribution of progression to high-grade cervical lesions.

The study was designed to identify clinically relevant progression events, with a focus on the histological diagnosis of CIN3 as the primary endpoint.

### 2.2. Study Population

Consecutive women treated at the Colposcopy Outpatient Clinic of the Department of Obstetrics and Gynecology, Catania University Hospital, between 2020 and 2025 were included in the study.

The final cohort consisted of 82 patients diagnosed with LSIL/CIN1, positive for high-risk HPV, and with adequate follow-up for outcome analysis.

Persistent LSIL was defined as at least two consecutive LSIL cytology results obtained at a minimum interval of 12 months, with documented HPV positivity and absence of CIN2+ at enrollment.

#### 2.2.1. Inclusion Criteria

Patients who met the following criteria were included: cytological diagnosis of LSIL at baseline; high-risk HPV positivity documented using a validated test; histological diagnosis of CIN1 if biopsy performed and conservative management; documentation of persistent low-grade condition and availability of clinical follow-up; availability of complete data regarding cytology, HPV testing, colposcopy, and any histological findings.

Baseline (time zero) was defined as the date of the second consecutive LSIL cytology, obtained at least 12 months after the initial LSIL diagnosis, confirming persistence and marking entry into the structured active surveillance program. Therefore, all included women had demonstrated at least 12 months of LSIL persistence prior to study inclusion.

#### 2.2.2. Exclusion Criteria

Patients were excluded with cytological evidence of HSIL or histological lesion ≥ CIN3 at baseline; prior excisional treatment; insufficient follow-up or incomplete data to define endpoints; clinical conditions requiring immediate treatment regardless of lesion grade.

### 2.3. Diagnostic Procedures and Follow-Up

Patients underwent an active surveillance program that included: cervicovaginal cytology; high-risk HPV testing with genotyping and colposcopy, performed by experienced colposcopists, with evaluation of the squamocolumnar junction, transformation zone, and colposcopic findings. In the presence of suspicious colposcopic findings or worsening cytological abnormalities, a targeted biopsy was performed.

In the Italian clinical context, women with HPV-positive LSIL are typically referred for colposcopic evaluation, and in cases of histologically confirmed CIN1, conservative management with structured surveillance is recommended. According to national and international recommendations, including those of the Italian Society of Colposcopy and Cervico-Vaginal Pathology (SICPCV) and the 2019 ASCCP risk-based management guidelines, excisional treatment may be considered after 24 months of persistent LSIL/CIN1, particularly when colposcopic assessment is unsatisfactory or follow-up reliability is uncertain [20]. In the present study, management was conducted within a tertiary referral university setting with structured follow-up and expert colposcopy. Clinical decisions were guided by a combination of cytological, virological, and colposcopic findings, according to a risk-based active surveillance approach.

HPV genotyping was performed on exocervical and endocervical samples collected in ThinPrep medium using the INNO-LiPA HPV Genotyping Extra II assay (Fujirebio Inc., Tokyo, Japan), which allows the identification of 28 viral genotypes. For analytical purposes, patients were stratified into two groups: HPV 16/18 and non-16/18 HPV, given the well-established higher oncogenic potential of the former.

### 2.4. Treatment Strategy

The therapeutic strategy adopted in the study was conservative and aimed at minimizing unnecessary treatments in low-risk patients. Specifically, excisional treatment was not performed solely for persistent LSIL cytology or CIN1 histology. CIN2 lesions were managed conservatively only in carefully selected cases, based on individualized clinical assessment including age, reproductive plans, adequacy of colposcopic evaluation, HPV genotype, and shared decision-making. Conservative management was not applied indiscriminately but within a structured surveillance program in a tertiary referral setting.

Excisional treatment (LEEP or conization) was reserved exclusively for patients with a histological diagnosis of CIN3. All histological diagnoses were reviewed by expert gynecologic pathologists in a tertiary referral center.

### 2.5. Outcome Definitions

#### 2.5.1. Primary Outcome

The primary outcome was progression to CIN3, defined as histologically confirmed cervical intraepithelial neoplasia grade 3 based on targeted biopsy or surgical specimens (LEEP or conization).

#### 2.5.2. Secondary Outcome

Secondary outcomes included clearance of HPV infection (HPV test negativization), with or without cytological normalization; persistence of HPV positivity and/or LSIL cytology; CIN2 diagnosis during follow-up; and time to progression to CIN3.

### 2.6. Duration of Follow-Up

Follow-up was calculated from baseline until the diagnosis of the event of interest (CIN3) or until the last available visit in the absence of progression. The median follow-up duration was 48 months, with a range between 24 and 78 months.

### 2.7. Statistical Analysis

Statistical analysis was performed to describe the clinical course of patients under active surveillance for persistent LSIL and to evaluate the association between HPV16 status and the risk of progression to high-grade lesions.

All analyses were conducted using R 4.5.2 software (R Foundation for Statistical Computing, Vienna, Austria). Statistical tests were two-sided, and a *p* value < 0.05 was considered statistically significant.

Continuous variables were summarized as median and range (minimum–maximum), while categorical variables were reported as absolute frequencies and percentages.

#### 2.7.1. Endpoint and Event Definition

The primary endpoint was histologically confirmed progression to CIN3 during follow-up. Time to event was calculated in months from the date of inclusion in the active surveillance program to the date of CIN3 diagnosis. Patients who did not develop CIN3 were censored at the date of the last available visit (end of follow-up). Follow-up duration was reported as median (range).

#### 2.7.2. Survival Analysis (Kaplan–Meier)

Survival analysis was performed using the Kaplan–Meier method to estimate the cumulative probability of remaining free from CIN3 over time. The CIN3-free survival curve was generated for the entire cohort. Results were graphically displayed, including the number of patients at risk at each time point and the 95% confidence intervals (CIs).

#### 2.7.3. Stratified Analysis by HPV16 Status and Comparison of Survival Curves

Kaplan–Meier curves were subsequently stratified according to HPV16 status (HPV16 vs. non-HPV16) to explore differences in the time-dependent risk of progression. Survival curves were compared using the log-rank test, with a *p* value < 0.05 considered statistically significant.

#### 2.7.4. Cox Model (Hazard Ratio)

To estimate the association between HPV16 and the risk of progression to CIN3, a Cox proportional hazards regression model was applied, reporting the hazard ratio (HR) with its 95% CI and *p*-value. The discriminative accuracy of the model was described by the concordance index (C-index). Given the limited number of CIN3 events, Cox regression estimates should be interpreted cautiously and are presented primarily for exploratory purposes.

#### 2.7.5. Assessment of Proportional Hazards Assumption (Schoenfeld Residuals)

The proportional hazards assumption of the Cox model was assessed using the Schoenfeld residuals test. In cases where non-proportionality was detected (global test *p* < 0.05), Cox model estimates were interpreted with caution, and the risk pattern was primarily described using Kaplan–Meier analysis and the temporal distribution of events.

#### 2.7.6. Descriptive Analysis of Early Time Window (0–24 Months) (Landmark Approach)

Given the early concentration of events observed in the cohort, a descriptive analysis of progression timing was performed, distinguishing between events occurring within 24 months and those occurring after 24 months (Landmark approach). This assessment was used to characterize a potential early risk window during surveillance.

## 3. Results

**Figure 1:**
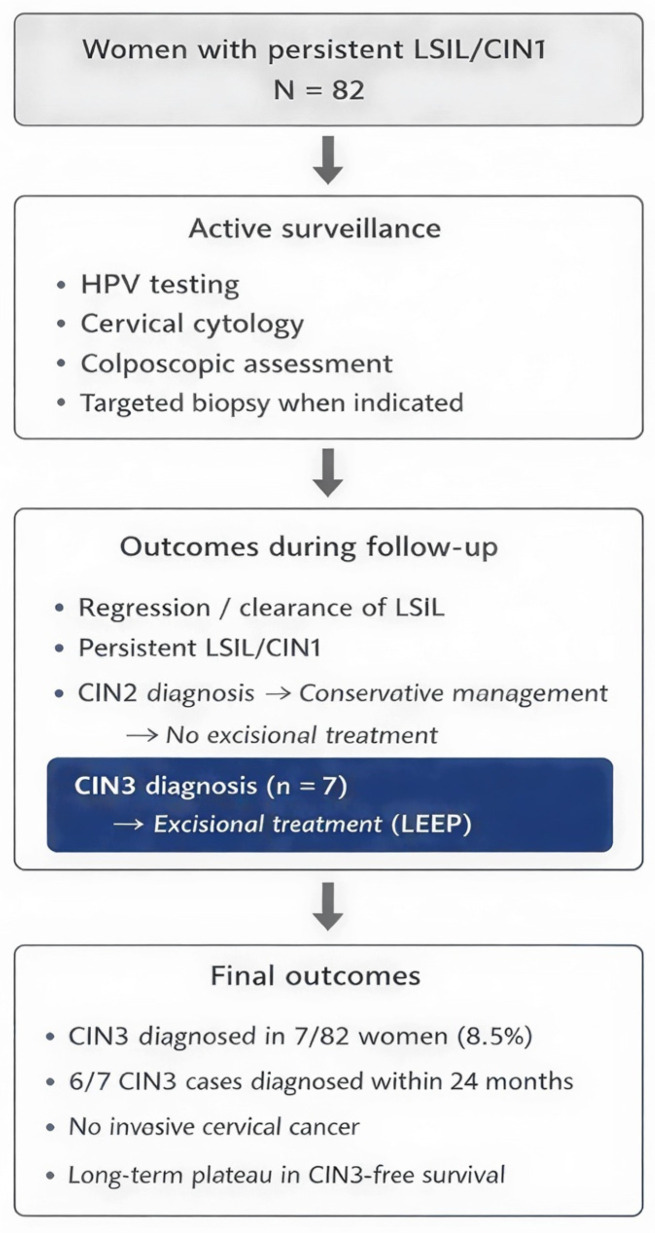
Patient selection, surveillance strategy, and clinical management during follow-up are summarized in the study flow-chart.

The study included a total of 82 women with a cytological diagnosis of persistent HPV-positive LSIL, undergoing active surveillance at the Colposcopy Center of the Obstetrics and Gynecology Unit of the Catania Hospital (Figure 1).

All patients were ≥25 years of age and had at least two consecutive cytology reports of LSIL at least 12 months apart. Within the cohort, 35 patients (42.7%) tested positive for HPV 16, while 47 patients (57.3%) had no HPV 16 genotypes.

The follow-up, with longitudinal observation up to 72 months and a median duration of 48 months, allowed for a reliable assessment of medium- to long-term clinical evolution.

During the follow-up, the distribution of clinical and histological outcomes in the study population was as follows: 7 cases of CIN3, equal to 8.5%; 2 cases of CIN2, equal to 2,4%; 25 cases of CIN1, equal to 30.5%; and 47 cases of clearance, equal to 57.3%. No cases of invasive cervical carcinoma were observed during the observation period.

Histological progression to CIN3, the primary outcome of the study, was observed in seven patients. All CIN3 cases were diagnosed during follow-up and were the only indication for excisional treatment with LEEP.

The two patients diagnosed with CIN2 were managed with active surveillance, without resorting to excisional treatment, in accordance with the conservative approach adopted in the study. None of the CIN2 cases showed immediate progression to CIN3 during the available follow-up.

In the HPV16-positive subgroup (n = 35), outcomes observed during follow-up included 5 cases of CIN3, 2 cases of CIN2, a notable proportion of low-grade lesions (CIN1), and cases of viral clearance (Table 1).

In the HPV16-negative subgroup (n = 47), progression to CIN3 was less frequent, with only 2 cases, while most patients exhibited viral clearance or persistence of low-grade lesions.

Of particular interest, the temporal analysis of events showed that Kaplan–Meier survival analysis of the entire cohort revealed a high probability of remaining free from CIN3 during follow-up, with a decline in the curve concentrated primarily in the early observation period, followed by subsequent stabilization.

Indeed, progression to CIN3 showed a marked early concentration, with 6 out of 7 cases (85.7%) diagnosed within the first 24 months of surveillance, while only one additional event was observed beyond 24 months; no late progressions were observed beyond this interval, despite extended follow-up of up to 72 months in some patients.

This trend suggests the existence of a predominantly early window of risk, followed by a subsequent phase characterized by substantial stability of risk and a plateau in the probability of remaining free of CIN3 over the long term.

The stratified analysis highlighted a clear difference in the distribution of events between the two groups.

In the HPV16-positive group (n = 35), progression to CIN3 was observed in 5 patients (approximately 14.3%). All these cases occurred early, within the first 24 months of follow-up. In the same group, 2 diagnoses of CIN2 were also observed, both detected within 24 months, with no further cases in subsequent follow-ups.

**Figure 2:**
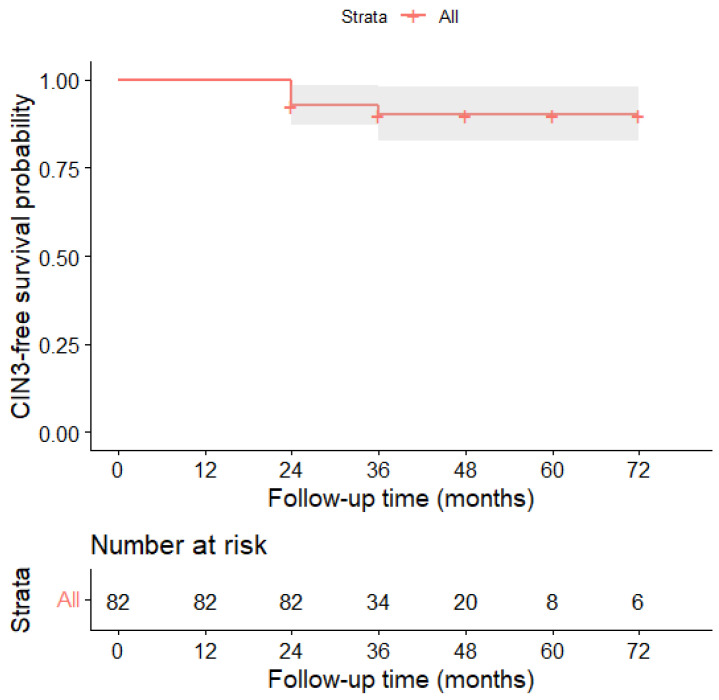
Overall Kaplan–Meier curve for CIN3-free survival. Kaplan–Meier estimate of CIN3-free survival in the overall cohort of women with persistent LSIL/CIN1 managed with active surveillance. Shaded areas represent the 95% confidence interval (CI). The number of patients at risk is reported below the plot.

**Figure 3:**
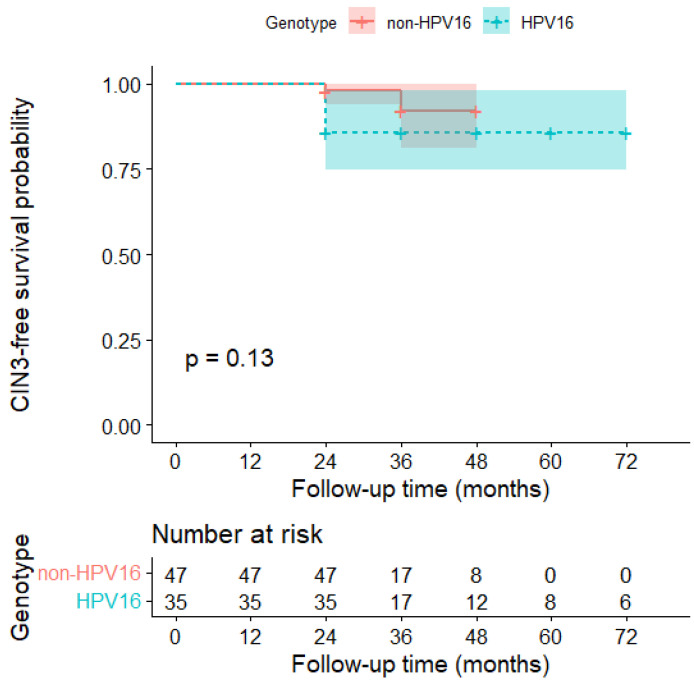
Kaplan–Meier curves for CIN3-free survival stratified by HPV16 status. Kaplan–Meier estimates of CIN3-free survival stratified by HPV16 status (HPV16 vs. non-HPV16). Shaded areas represent the 95% confidence interval (CI). The log-rank test was used to compare survival curves (*p* = 0.13). The number of patients at risk is reported below the plot.

In the HPV16-free group (n = 47), progression to CIN3 was less frequent, with two cases overall (approximately 4.3%). In this group, progression had a slightly more diluted temporal distribution than in the HPV16 group, with one case within 24 months and one case within 36 months, and no events beyond that period (Figure 2 and Figure 3).

These results indicate that HPV16 positivity showed a higher observed incidence of progression to CIN3 and an earlier temporal clustering of events, although statistical significance was not reached.

Kaplan–Meier curves suggested a higher and earlier risk in the HPV16-positive group; however, comparison of the curves using the log-rank test did not reach statistical significance (χ^2^ = 2.3, *p* = 0.10), although the trend was consistent with a higher incidence of CIN3 in HPV16-positive patients (HR 3.38, 95% CI 0.65–17.47) (Table 2).

In the Cox regression model, HPV16 positivity showed a trend toward an increased hazard of progression to CIN3, although the estimate was imprecise and did not reach statistical significance (*p* = 0.146) (Table 2).

Assessment of the proportional hazards assumption using Schoenfeld residuals showed borderline evidence of non-proportional hazards (global test *p* = 0.049), suggesting a possible variation in the effect of HPV16 over time (Table 3). However, given the very limited number of outcome events, this finding should be interpreted with caution, as proportional hazards tests may be unstable in small samples.

A Cox regression analysis using a 24-month Landmark approach was performed to account for the early concentration of events. During the 0–24 month period (N = 82), six cases of progression to CIN3 occurred. HPV16 positivity was associated with a markedly increased risk of CIN3 compared with the HPV16-negative group (HR 7.09, 95% CI 0.83–60.66). The association showed borderline significance by the Wald test (*p* = 0.074) and was supported by the likelihood ratio and score tests (*p* = 0.03 and *p* = 0.04, respectively) (Table 4).

Overall, the results indicate that progression to CIN3 in women with persistent LSIL is a rare event, occurs mainly within the first two years of follow-up, and tends to be more frequent in HPV16-positive patients, although the limited number of events reduces the statistical power of the estimates.

### 3.1. Clearance and Persistence

In the HPV16-positive group, clearance was observed in 13 patients within 24 months, with additional clearances documented at subsequent follow-ups (5 at 36 months, 4 at 48 months, 2 at 60 months, and 2 at 72 months), suggesting that even in the presence of HPV16, a significant proportion of patients may become negative over time.

In the non-HPV16 group, clearance was numerically more frequent in the early stages of follow-up, with 29 patients showing negative results within 24 months and further clearances at 24 and 36 months.

Concurrently, the persistence of LSIL was observed in a significant proportion of patients in both groups, without this persistence translating into a progressive increase in risk over time, given that CIN3 events were concentrated exclusively within the first 24 months.

### 3.2. Histology of Progressive Cases and Treatment

In cases that reached the primary endpoint (CIN3), excisional treatment was performed according to the study protocol, reserved only for clinically relevant progression. In all treated cases, histological analysis of the surgical specimen revealed the coexistence of CIN1 and CIN3 lesions, confirming the simultaneous presence of alterations of varying degrees in the same cervix.

## 4. Discussion

This study provides insight into the natural history of persistent HPV-positive LSIL in a cohort of women managed with active surveillance, where excisional treatment was reserved exclusively for cases showing histological progression to CIN3. Our findings indicate that, in this clinical setting, persistent LSIL is associated with a relatively low risk of clinically significant progression, with most women experiencing a benign or regressive course over time.

Although LSIL is generally considered a low-grade lesion, evidence from large screening cohorts has shown that HPV-positive LSIL cytology is associated with a measurable risk of high-grade disease. In particular, Katki et al. reported a 5-year risk of CIN2+ of 19% and CIN3+ of 6.1% among women aged 30–64 years with HPV-positive LSIL cytology. However, our study differs from screening-based analyses because it specifically focuses on women with documented persistent HPV-positive LSIL who were managed within a structured surveillance program [21].

One of the most relevant findings of our study is the clear temporal clustering of progression events. All CIN3 cases occurred within the first 24 months of follow-up, and no late events were observed beyond this interval despite extended observation of up to five years in a substantial proportion of patients. This pattern suggests that the risk associated with persistent LSIL is not cumulative over time but rather concentrated during the early phase of surveillance.

This temporal pattern is consistent with evidence from large cohort studies showing that the risk of high-grade cervical lesions is primarily driven by early persistence of high-risk HPV infection rather than by the duration of low-grade cytological abnormalities.

Within the broader context of long-term risk assessment in low-grade cervical disease, recent population-based studies provide additional insight for interpreting our findings. Baasland et al. recently reported national long-term data from more than 26,000 women with histologically confirmed CIN1, demonstrating that although progression to CIN3+ remains relatively uncommon, treatment rates in routine clinical practice are nonetheless substantial [22]. While this large population-based study provides important epidemiological information, our cohort differs in several key aspects. Specifically, it includes exclusively women with documented persistent HPV-positive LSIL managed within a structured active surveillance program. Moreover, our analysis highlights the temporal distribution of progression events, showing a clear concentration of CIN3 cases during the early follow-up period rather than a cumulative increase in long-term risk.

Our findings are also consistent with population-based cohort evidence indicating that the risk of cervical precancer is primarily determined by early persistence of high-risk HPV infection rather than by the duration of low-grade cytological abnormalities. In the landmark cohort study by Castle et al., excess risk of CIN3+ emerged early after HPV persistence, particularly for HPV16, without increasing linearly over time [23]. Similarly, in our cohort, progression to CIN3 was concentrated within the first 24 months of surveillance, supporting a time-dependent risk model.

Additional evidence comes from a Norwegian follow-up study by Sørbye et al., which reported similar risks of CIN2+ among women with an initially negative biopsy and those with CIN1 [19]. Taken together, these observations support the concept that virological and cytological markers may provide more informative risk stratification than the presence of a low-grade histological lesion alone.

An important biological aspect emerging from the interpretation of our results concerns the relationship between LSIL and high-grade lesions [24]. Traditionally, LSIL has been considered part of a linear continuum of cervical carcinogenesis.

In this context, the occurrence of CIN3 during follow-up of persistent LSIL does not necessarily reflect linear biological progression of the same lesion. Instead, it may represent delayed detection of an initially unrecognized high-grade lesion or the de novo development of a transformative lesion associated with specific virological determinants. This interpretation is supported by virological studies showing that CIN1 and CIN3 lesions within the same cervix may be associated with different HPV genotypes, suggesting distinct biological origins. Park et al. demonstrated that lesions of different grades may arise independently, and Lityens et al. further reported that CIN3 lesions are rarely preceded by CIN1 and that the presence of LSIL alone does not intrinsically increase the risk of subsequent HSIL [13,25,26].

Our findings are consistent with this biological paradigm, suggesting that the risk of CIN3 appears more closely associated with persistence of high-risk HPV genotypes, particularly HPV16, 31, 33, and 18, than with the simple presence of CIN1 [27,28,29]. In this context, viral genotype plays a central role in risk stratification. In our cohort, progression to CIN3 was more frequent among HPV16-positive women compared with HPV16-negative women, showing a tendency toward earlier progression, although this difference did not reach statistical significance in the log-rank test (*p* = 0.13) [30,31]. This trend is clinically consistent with the well-established oncogenic potential of HPV16 and its predominant role in cervical carcinogenesis.

These considerations have important clinical implications. If LSIL predominantly represents the expression of a productive HPV infection rather than a true precancerous lesion, persistence of the lesion over time should not automatically be interpreted as an indication for excisional treatment. Instead, a risk-stratified approach integrating virological, cytological, and colposcopic factors may allow more appropriate patient management, reducing unnecessary procedures while preserving cervical integrity. Our findings are consistent with the risk-based management paradigm promoted by the 2019 ASCCP and 2023 ESGO guidelines, which have moved beyond static decision-making based solely on cytological diagnosis or lesion duration. These guidelines emphasize that persistence of LSIL, in the absence of additional risk indicators, does not automatically justify excisional treatment but requires individualized risk assessment based on the estimated probability of CIN3+.

Although CIN2 lesions were observed during follow-up and showed regression, CIN2 was not considered a study endpoint. This decision reflects the growing recognition of the biological heterogeneity and high regression rate of CIN2, particularly when occurring in the context of LSIL, as demonstrated in the ALTS study, and supports reserving excisional treatment exclusively for confirmed CIN3 cases [32,33,34,35,36].

This study has several limitations. Its retrospective nature may introduce inherent selection bias. The single-center design and the conduct of the study in a tertiary academic setting with experienced colposcopists and centralized histopathological review may limit generalizability to community-based screening settings, where clinical expertise and follow-up adherence may differ. The cohort represents women with persistent LSIL who had not progressed during the first year of observation, potentially introducing a form of survival bias and limiting generalizability to all newly diagnosed LSIL cases.

Assessment of proportional hazards in the Cox model indicated a borderline signal of possible time-varying effects for HPV16. However, given the limited number of outcome events, this finding should be interpreted cautiously, as formal proportional hazards tests may be unstable in small samples. Therefore, the temporal distribution of events and descriptive Kaplan–Meier analyses were considered the most reliable representation of risk patterns in this cohort.

## 5. Conclusions

In conclusion, persistent HPV-positive LSIL appears to represent a low-risk clinical condition when managed within a structured surveillance program. Progression to CIN3 is limited and largely concentrated in the early phases of follow-up, suggesting that risk is more strongly determined by virological factors than by the simple persistence of low-grade histology. Taken together, these findings support a risk-based management approach in women with persistent HPV-positive LSIL, in which structured surveillance represents a safe strategy for most patients and contributes to reducing unnecessary treatments.

## Figures and Tables

**Table 1:** Cumulative incidence of CIN3 during follow-up according to HPV16 status.

Time (Months)	HPV16 (n = 35)—CIN3 Cumulative n (%)	Non-HPV16 (n = 47)—CIN3 Cumulative n (%)	Total CIN3 n (%)	Patients at Risk
12	3 (8.6%)	1 (2.1%)	4 (4.9%)	82
24	5 (14.3%)	1 (2.1%)	6 (7.3%)	78
36	5 (14.3%)	2 (4.3%)	7 (8.5%)	34
48	5 (14.3%)	2 (4.3%)	7 (8.5%)	20
60	5 (14.3%)	2 (4.3%)	7 (8.5%)	8
72	5 (14.3%)	2 (4.3%)	7 (8.5%)	6

Values represent cumulative numbers of histologically confirmed CIN3 cases at each time point. “Patients at risk” indicates the number of women still under follow-up and free from CIN3 at the beginning of each interval. The reduction over time reflects censoring due to follow-up completion or loss to follow-up.

**Table 2:** Log-rank test and Cox proportional hazards model for progression to CIN3 stratified by HPV status.

Group	N	Events CIN3 n (%)	Log-Rank χ^2^ (df = 1)	*p*-Value (log-Rank)	HR Cox (HPV16 vs. Non-HPV16)	95% CI	*p*-Value Cox
non-HPV16	47	2 (4.3%)			Ref		
HPV16	35	5 (14.3%)	2.30	0.13	3.38	0.65–17.47	0.146
Total	82	7 (8.5%)	2.30	0.10	—	—	—

**Table 3:** Schoenfeld residuals test for proportional hazards assumption in the Cox regression model.

Variable	Chi-Square	df	*p*-Value
HPV16 status	3.86	1	0.049
Global test	3.86	1	0.049

**Table 4:** Landmark Cox regression analysis at 24 months for progression to CIN3 according to HPV16 status.

Time Window	N	Events	Predictor	HR	95% CI	Wald *p*-Value	LR Test *p*-Value	Score Test *p*-Value
0–24 months	82	6	HPV16 (vs. non-HPV16)	7.09	0.83–60.66	0.074	0.03	0.04

Abbreviations: HR, hazard ratio; CI, confidence interval; LR, likelihood ratio. Note: A 24-month landmark was selected due to the early clustering of CIN3 events.

## Data Availability

The data are contained within the article.
